# Trends in Care and Treatment for Persons Aged ≥13 Years with HIV Infection 17 U.S. Jurisdictions, 2012-2015

**DOI:** 10.2174/1874613601812010090

**Published:** 2018-09-28

**Authors:** Debra L. Karch, Xueyuan Dong, Jing Shi, H. I. Hall

**Affiliations:** 1 HIV Incidence and Case Surveillance Branch, Division of HIV Prevention, National Center for HIV/AIDS, Viral Hepatitis, STD and TB Prevention, Centers for Disease Control and Prevention, 1600 Clifton Road NE, MS E-47, Atlanta, GA 30329, U.S; 2 ICF International, Inc, Atlanta, GA, U.S

**Keywords:** HIV Infection, Trend, Linkage, Treatment, Viral Suppression, CD4

## Abstract

**Background:**

Care and viral suppression national goals for HIV infection are not being met for many at-risk groups. Assessment of the trends in national outcomes for linkage to care, receipt of care, and viral suppression among these groups is necessary to reduce transmission.

**Methods:**

Data reported to the National HIV Surveillance System by December 2016 were used to identify cases of HIV infection among persons aged 13 years and older in one of 17 identified jurisdictions with complete laboratory reporting. We estimated national trends in HIV-related linkage to care, receipt of care and viral suppression using estimated annual percent change from 2012-2015 for various characteristics of interest, overall and stratified by sex and race/ethnicity.

**Results:**

Overall, trends in linkage to and receipt of care and viral suppression increased from 2012-2015. Generally, linkage to and receipt of care increased among young black and Hispanic/Latino males, those with infection attributed to male-to-male sexual contact, and those not in stage 3 [AIDS] at HIV diagnosis. All sub-groups showed improvement in viral suppression. Within years, there remains a substantial disparity in receipt of care and viral suppression among racial/ethnic groups.

**Conclusion:**

While trends are encouraging, scientifically proven prevention programs targeted to high-risk populations are the foundation for stopping transmission of HIV infection. Frequent testing to support early diagnosis and prompt linkage to medical care, particularly among young men who have male to male sexual contact, black and Hispanic/Latino populations, are key to reducing transmission at all stages of disease.

## 1 INTRODUCTION

In the United States, between 2010 and 2014 the rate of people living with HIV steadily increased from 275.7/100,000 population to 299.5/100,000 population, respectively. In 2010, the rate of HIV diagnoses was 14.2/100,000 population and the rate declined to 12.3/100,000 population in 2015 [1]. The highest rates of new diagnoses in 2015 were among blacks/African Americans (hereafter referred to as blacks) (44.3/100,000 population), more specifically black males (84.8/100,000 population), blacks aged 20-24 years (111.2/100,000 population) and blacks 25-29 years (112.2/100,000 population) [1]. Hispanics/Latinos also experienced elevated rates of HIV diagnoses over whites although the rates remained at approximately one-third to one-half the rates of blacks with the same sex and age characteristics. The vast majority of males with HIV infection diagnosed in 2015 had infection attributed to male-to-male sexual contact or male-to-male sexual contact in conjunction with injection drug use (86.2%); females had infection attributed primarily to heterosexual contact (86.3%) [1]. For these and other risk groups, prevention services are essential to avoid further spread of HIV infection and, for those living with HIV infection, coordinated supportive services and medical care are crucial [2].

Goals of the National HIV/AIDS strategy for the United States: Updated to 2020 [3] include reducing new HIV infections, increasing access to care and improving health outcomes for people living with HIV infection, reducing HIV-related disparities and health inequities, and achieving a more coordinated national response to the HIV epidemic. Progress toward these goals by 2020 includes: increasing to 85% the number of newly diagnosed persons who are linked to HIV medical care within one month of diagnosis, increasing to 90% the number of persons living with diagnosed HIV who are retained in HIV medical care, increasing to 80% the number of persons diagnosed with HIV who have suppressed viral load, and reducing disparities by focusing on high risk groups comprised of young black gay and bisexual males and black females [4] for whom disparities in HIV diagnoses and treatment outcomes have been demonstrated [5-8].

Data from the National HIV Surveillance System, described elsewhere [9, 10], show that for people with HIV infection diagnosed in 2015 in 37 states and the District of Columbia, 75.0% were linked to care within one month of diagnosis and for those with HIV diagnosed by year-end 2013 and alive at year-end 2014, 72.5% were retained in care and 57.9% had suppressed viral load [11]. All three indicators fall short of national goals. In 2014, for 32 states and the District of Columbia, outcomes for these three indicators were even lower for black males (69.9%, 67.0%, and 47.9% respectively), males aged 18-24 years (66.2%, 71.4%, and 44.4% respectively), and people who inject drugs (74.3%, 64.3%, and 47.1% respectively) [12].

Assessing changes in linkage to care, received care, and viral suppression over the last decade has been hindered by a lack of comprehensive HIV laboratory reporting laws that require reporting of all CD4 results regardless of value and all viral load results including detectable and undetectable; and by incomplete laboratory reporting within states with comprehensive reporting laws [13]. This study examined annual changes for 2012 to 2015 in linkage to care, receipt of care, and viral suppression among adults and adolescents using data reported for 17 jurisdictions to the National HIV Surveillance System. These jurisdictions represented approximately 46% of the U.S. population. We aimed to explore these changes by patient characteristics, stage of HIV infection at diagnosis and transmission category.

## 2 METHODS

Data reported to the National HIV Surveillance System by December 2016 were used to identify cases of HIV infection that met the CDC HIV infection case definition [14] among persons aged 13 years and older at time of diagnosis and whose residence at diagnosis (for linkage to care) and most current residence (for receipt of care and viral suppression) were within one of 17 identified jurisdictions with complete laboratory reporting. To be eligible for inclusion jurisdictions were required to meet the following three criteria for each year between 2012 and 2015:

- The jurisdiction’s laws/regulations required the reporting of all CD4 and viral load results to the state/city health department,
- Laboratories that perform HIV-related testing for the areas must have reported a minimum of 95% of HIV-related test results to the state/city health department, and
- By December 31, 2016, the area had reported to CDC at least 95% of all CD4 and viral load test results received from January 2012 through December 2015.

The jurisdictions that met inclusion criteria were California, District of Columbia, Hawaii, Iowa, Illinois, Indiana, Louisiana, Maryland, Michigan, Missouri, New Hampshire, New York, North Dakota, South Carolina, Texas, Utah, and West Virginia.

Linkage-to-care analysis included people with HIV infection diagnosed between January 1^st^ and December 31^st^ of the outcome year (*e.g.*, the outcome for 2012 includes all diagnoses between January 1, 2012 and December 31, 2012). Linkage to care was defined as ≥1 CD4 or viral load test performed within 1 month of diagnosis.

Receipt of care and viral load suppression analyses included people with HIV diagnosed before January 1^st^ of the outcome year and not known to be deceased on December 31^st^ of the outcome year (*e.g.*, the result for 2012 includes all persons with an HIV diagnosis before January 1, 2012 and not known to be deceased on December 31, 2012). Receipt of care was defined as ≥1 CD4 or viral load test performed during the outcome year. Viral suppression was defined as a viral load result of <200 copies/mL or, if the quantitative value was missing, a test interpretation value of “undetected”, at the time of the most recent viral load test during the outcome year.

Laboratory results with missing month or year of specimen collection were excluded from the analysis (<0.36% for linkage-to-care and <0.16% for receipt of care and viral suppression). All duration times were calculated using the month and year for both HIV infection diagnosis and laboratory results. If a patient had two tests in the same month with different viral suppression results, we applied a conservative approach and used the test result that indicated a higher viral load. In addition, laboratory tests with a missing result and tests with specimen collection dates prior to the date of HIV infection diagnosis were excluded.

The twelve-month interval between the last observation year (2015) and dataset used (reporting through December 2016) allowed time for reporting of diagnoses, laboratory results, and deaths. Data were adjusted for unknown or missing transmission category [15]. Results are presented by age group (13-24, 25-34, 35-44, 45-54, ≥55), sex, race/ethnicity (black, Hispanic/Latino, white, and other) (those with missing race/ethnicity were excluded from the analysis), and transmission category (male-to-male sexual contact, people who inject drugs, male-to-male sexual contact and injection drug use, heterosexual contact, and other). Stage of disease at diagnosis was categorized as diagnosis of HIV-infection Stage 3 [AIDS] within 3 months of an HIV diagnosis, or the absence of HIV-infection Stage 3 [AIDS] diagnosis within 3 months of an HIV diagnosis. The estimated annual percent change (EAPC) was calculated for each person characteristic and considered statistically significant at *P*-value <.05. All analyses were conducted using SAS 9.3 (SAS Institute, Inc., Cary, NC). This project was approved by CDC as a retrospective, secondary data analysis using HIV surveillance data. This analysis did not constitute research involving identifiable human subjects requiring IRB review.

## 3 RESULTS

Linkage to care among the 81,174 (2012=20,876; 2013=19,887; 2014=20,467; and 2015=19,944) people with HIV infection diagnosed between January 1, 2012 and December 31, 2015 increased each year from 2012-2015 (79.9%, 81.4%, 82.9%, and 83.7% respectively, EAPC = 1.6, 95% CI 0.9-2.3, *P*<.001) (Table **1**). In 2015, linkage to care was higher among whites compared with all other races/ethnicities, older age groups compared with younger age groups, and those with HIV-infection Stage 3 [AIDS] compared with those without stage 3 [AIDS] diagnosis. Linkage was also higher among women and those with infection attributed to heterosexual contact. Linkage to care increased significantly from 2012-2015 among males (EAPC=1.6, 95% CI 0.9-2.4, *P*<.001), blacks (EAPC=2.2, 95% CI 1.1-3.3, *P<*.001), those with HIV diagnosed earlier than stage 3 (AIDS) (EAPC=2.4, 95% CI 1.6-3.2, *P*<.001), males with infection attributed to male-to-male sexual contact (EAPC=1.6, 95% CI 0.7-2.4, *P*<.001), those aged 13-24, 25-34, and 35-44 years at diagnosis (EAPC=3.2, 95% CI 1.7-4.7, *P*<.001, EAPC=1.3, 95% CI 0.1-2.5, *P*=.039, and EAPC=1.7, 95% CI 0.2-3.2, *P*=.031 respectively), and those with a transmission category of heterosexual contact (EAPC=1.8, 95% CI 0.4-3.3, *P*=.014).

By race/ethnicity and sex, linkage to care increased among black males overall (EAPC=2.5, 95% CI 1.2-3.8, *P*<0.001), and specifically among black males aged 13-24 years (EAPC=3.4, 95% CI 1.1-5.8, *P*=0.004), those in earlier than stage 3 of the disease at diagnosis (EAPC=3.8, 95% CI 2.2-5.3, *P*<0.001) and those with infection attributed to male-to-male sexual contact (EAPC=2.4, 95% CI 1.0-3.9, *P*=0.001) (Table **2**). Black females showed no significant improvements in linkage to care from 2012-2015 with the exception of those with HIV-infection diagnosed without stage 3 [AIDS] (*P=*0.045). Similarly, linkage to care among Hispanic/Latino males aged 13-24 years (EAPC=3.2, 95% CI 0.3-6.2, *P*=0.033) and those with HIV infection without a diagnosis of stage 3 [AIDS] (EAPC=2.1, 95% CI 0.5-3.8, *P*=0.010) increased. There were no significant increases in linkage to care from 2012-2015 among Hispanic/Latino females or white males or females.

Overall, the proportion of people with diagnosed HIV infection who received care increased from 2012 to 2015 (EAPC=0.6, 95% CI 0.4-0.8, *P*<0.001) (Table **3**). Receipt of care increased significantly for all individual categories of person characteristics assessed from 2012 to 2015, with the exception of those identifying as other race/ethnicity and other transmission category, those aged 45-54 years, and people who inject drugs. In each of the four years, the proportion of people who received care was consistently higher among whites and those classified as other race/ethnicity compared to blacks and Hispanics/Latinos and consistently lowest among those with a transmission category of injection drug use. There was little variation among age groups within years.

By race/ethnicity and sex, significant increases in the proportion of those who received care annually from 2012-2015 were found among black males of all ages except ≤55 years, Hispanic/Latino males aged 13-24 and 25-34 years, and all racial/ethnic groups diagnosed without Stage 3 [AIDS] except Hispanic/Latino and white females (Table **4**). Receipt of care increased from 2012-2015 among all racial/ethnic groups of men who have male to male sexual contact (blacks EAPC=1.0, 95% CI 0.6-1.4, *P*<.001; Hispanics/Latinos EAPC=0.8, 95% CI 0.4-1.2, *P*<.001; whites EAPC=0.5, 95% CI 0.1-0.8, *P*=.004). Receipt of care also increased among black females aged 35-44 years (EAPC=1.0, 95% CI 0.2-1.8, *P=*.020) and transmission category of heterosexual contact (EAPC=0.7, 95% CI 0.2-1.2, *P=*.008).

Viral suppression among people with diagnosed HIV infection increased annually from 2012 to 2015 overall (53.4%, 56.4%, 58.8%, and 59.2% respectively) (EAPC=3.5, 95% CI 3.3-3.7, *P*<.001) (Table **5**) and for all categories of person characteristics assessed. Within years, the proportion of people achieving viral suppression was consistently greater among males, whites, and people with HIV infection Stage 3 [AIDS], and consistently lowest among those with a transmission category of injection drug use. The proportion of those achieving viral suppression increased with age each year.

By race/ethnicity and sex, viral suppression increased annually from 2012-2015 for all characteristics except transmission category of other for all racial/ethnic groups, and Hispanic/Latino and white females aged 13-24 years (Table **6**).

## 4 DISCUSSION/IMPLICATIONS

This analysis explored progress in linkage to care, receipt of care, and viral suppression among people with HIV infection for outcome years 2012-2015 in 17 U.S. jurisdictions meeting the criteria for complete laboratory reporting for each of the four years. Linkage-to-care increases were found among some high-risk populations, particularly young people, blacks, males, and those engaging in male-to-male sexual contact. However, an increase was found in only one category of black women, those with a diagnosis of HIV Stage 3 [AIDS], despite black women being a priority population for HIV prevention. Notably, Hispanic/Latino and white females and white males showed no improvement in linkage to care from 2012-2015; however, the proportions at which they were linked to care were close to or higher than the national standard and exceeded linkage for black males by approximately five percentage points.

Generally, receipt of care improved among the same populations as linkage to care and also showed improvements for white men who have male to male sexual contact. Once again, no improvements were found among Hispanic/Latino or white women. Despite limited improvement in linkage to and receipt of care, and similar to another study [16], increases in viral suppression were seen in nearly every category of person characteristics assessed. This is potentially due to a number of factors including improvements in linkage to care and receipt of care that were found and increased prescribing of or compliance with antiretroviral therapy.

The distinction in linkage to and receipt of care and viral suppression between those with HIV diagnosed at stage 3 [AIDS] and not at stage 3 [AIDS] is prominent. Those with stage 3 [AIDS] are linked to care in greater proportions than those not in stage 3 [AIDS]. However, the differences between the two groups disappear for receipt of care. Then, once again, those with HIV diagnosed at Stage 3 [AIDS] achieve viral suppression more often than those not at stage 3 [AIDS]. These variations suggest the need to better understand why those with stage 3 [AIDS] are linked but did not receive care in any greater proportion than those not at stage 3 [AIDS].

A similar scenario is seen for linkage to and receipt of care and viral suppression by age group. While linkage to care and viral suppression both increase as age increases, this is not the case for receipt of care where the proportions vary within age categories. A better understanding of the impact of age on receipt of care could be important in increasing the proportion of those virally suppressed for all age groups.

Studies documenting the impact of poverty, poor education, substance use, mental health challenges, domestic violence, transportation to medical care, and lack of social support and employment on HIV care and treatment are abundant, and demonstrate disproportional impact on black men and women and Hispanics/Latinos, and on high-risk populations such as people who inject drugs and young men having sexual contact with men [7, 17-23]. Despite these barriers a number of evidence-based programs targeting these populations, including pre-exposure prophylaxis, demonstrate effective outcomes [24-26]. However, within years substantial differences remain in linkage to and receipt of care and viral suppression with whites exceeding blacks and Hispanics/Latinos, suggesting the effectiveness of programs to reduce the health disparities are limited [27].

The analysis was subject to several limitations. First, the outcomes assessed included only cases identified through 2015 and were under the guidance of the National HIV/AIDS Strategy for the United States: July 2010 [28]. The 2010 guidance established a linkage to care goal of 65% which was met for virtually every person characteristic group assessed and a retention in care goal of 80% for which our more liberal definition showed no person characteristic group met the goal. The 2010 guidance also set a goal of a 20% increase in the proportion of HIV diagnosed gay and bisexual men as well as Blacks with undetectable viral load which we cannot assess as we used 2012-2015 data. Improvements in testing and care and treatment since 2015 make it critical to continue to assess these outcomes under the new 2020 strategy released in July 2015. Second, the 17 jurisdictions may not be representative of all people with HIV infection in the United States. To mitigate the lack of representation we looked back no further than 2012. Including earlier years would have further reduced the number of states eligible for the analysis. Third, the EAPC for linkage to care is based on only four years of data due to the lack of complete laboratory reporting in other jurisdictions. Fourth, documentation of the most recent viral load may not be indicative of consistent viral suppression in this population over time [29] and further studies are needed to understand factors contributing to long term viral suppression. Fifth, exclusion of laboratory results with missing month or year of specimen collection date may underestimate linkage-to-care, receipt-of-care, and viral suppression. To address the majority of the limitation above, states continue to work with their legislatures to enact mandatory HIV-related laboratory test result reporting laws and all but six (Idaho, Kansas, New Jersey, Pennsylvania, Vermont, and the Virgin Islands) have now done so with the latest being Arizona in 2018. Opportunities to expand analyses will occur as states collect this data. Additional studies are needed as more jurisdictions begin to meet the laboratory reporting requirements.

## CONCLUSION

Improving care and treatment for people with HIV infection and reducing HIV-related disparities across the three indicators studied show some promising results; however, linkage-to-care and viral suppression indicators fall short of National HIV/AIDS Strategy for the United States: Updated to 2020 goals. While not a national indicator, the more inclusive definition used in this study for receipt of care (one HIV-related medical visit per year) still falls short of the retention in care (two or more HIV-related medical visits more than three months apart in a year) national goal and thus there also remain opportunities for improvement in receipt of care.

Prevention programs that are scientifically proven, cost-effective, scalable and targeted to high-risk populations are the foundation for stopping transmission of HIV infection. Frequent testing to support early diagnosis and prompt linkage to medical care, particularly among young men who have male to male sexual contact and black and Hispanic/Latino populations, are key to reducing transmission at all stages of disease, and specifically among those with acute infection when transmission risk is high. The substantial disparity in receipt of care and viral suppression among racial/ethnic groups suggests the need for improved targeting of interventions based on social determinants of health. Resources are still needed to monitor and improve outcomes across the HIV continuum of care. The transition from the July 2010 national strategy to the 2020 updated national strategy presents an opportunity to reassess local, state, and national programs to reach these care continuum goals.

## Figures and Tables

**Table 1:** Linkage to care among persons aged ≥13 years with HIV diagnosed during 2012-2015, by selected characteristics -- 17 US jurisdictionsa

**Characteristic**	**Total**	**Linked to care^b^ for persons diagnosed in the year**									**Trends from 2012 - 2015**		
		**2012**		**2013**		**2014**		**2015**		**EAPC**	**95% CI**		***P*-value**
		**(N=20,876)**		**(N=19,887)**		**(N=20,467)**		**(N=19,944)**					
		**No.**	**(%)**	**No.**	**(%)**	**No.**	**(%)**	**No.**	**(%)**		**Lower**	**Upper**	
**Sex**													
Male	66,312	13,464	79.6	13,150	81.0	13,822	82.5	13,693	83.5	1.6	0.9	2.4	<.001
Female	14,862	3,206	81.1	3,047	83.3	3,137	84.5	3,002	84.8	1.5	-0.1	3.1	0.068
**Race/Ethnicity**													
Black/African American	33,116	6,499	75.8	6,398	78.2	6,686	80.5	6,506	80.8	2.2	1.1	3.3	<.001
Hispanic/Latino	22,751	4,577	80.1	4,511	82.6	4,807	82.5	4,802	83.6	1.3	0.0	2.6	0.055
White	19,825	4,408	85.7	4,154	85.7	4,285	86.3	4,280	87.9	0.8	-0.5	2.2	0.228
Other	5,482	1,186	81.9	1,134	81.6	1,181	86.3	1,107	86.7	2.3	-0.3	5.0	0.087
**Age group at diagnosis**													
13-24	18,434	3,504	74.3	3,484	77.8	3,711	78.4	3,710	82.3	3.2	1.7	4.7	<.001
25-34	26,026	5,056	79.4	5,012	80.2	5,474	82.4	5,556	82.2	1.3	0.1	2.5	0.039
35-44	16,418	3,510	80.7	3,371	83.7	3,525	84.5	3,286	84.9	1.7	0.2	3.2	0.031
45-54	13,057	3,045	84.3	2,759	84.7	2,683	86.1	2,642	86.1	0.8	-0.9	2.5	0.345
55+	7,239	1,555	84.9	1,571	83.8	1,566	87.0	1,501	86.6	1	-1.3	3.2	0.406
**Stage at diagnosis**													
HIV infection stage 3(AIDS)	18,072	4,805	98.6	4,471	98.8	4,429	99.1	4,177	99.4	0.3	-1.0	1.6	0.693
Not known to be HIV infection stage 3	63,102	11,865	74.1	11,726	76.3	12,530	78.3	12,518	79.5	2.4	1.6	3.2	<.001
**Transmission Category^c^**													
Male-to-male sexual contact	55,276	11,138	79.7	10,904	81.2	11,677	82.7	11,490	83.5	1.6	0.7	2.4	<.001
Injection drug use	5,010	1,054	77.4	934	77.2	904	79.9	1,046	80.1	1.3	-1.4	4.1	0.341
Male-to-male sexual contact and injection drug Use	2,646	596	81.1	533	79.4	530	82.2	504	84.7	1.6	-2.2	5.5	0.422
Heterosexual contact**^d^**	18,068	3,842	80.7	3,787	83.4	3,821	84.5	3,622	85.3	1.8	0.4	3.3	0.014
Other**^e^**	174	40	78.5	39	83.9	27	69.0	33	84.8	0.5	-13.4	16.5	0.952
**Total**	**81,174**	**16,670**	**79.9**	**16,197**	**81.4**	**16,959**	**82.9**	**16,695**	**83.7**	**1.6**	**0.9**	**2.3**	**<.001**

Abbreviations: HIV= Human Immunodeficiency Virus, EAPC=Estimated Annual Percent Change. Note: The denominator for each characteristic for each year are not presented. ^a^Jurisdictions include California, District of Columbia, Hawaii, Iowa, Illinois, Indiana, Louisiana, Maryland, Michigan, Missouri, New Hampshire, New York, North Dakota, South Carolina, Texas, Utah, and West Virginia. ^b^Defined as one or more CD4+ T-lymphocyte or viral load test within 1 month after HIV diagnosis. ^c^Data statistically adjusted to account for missing transmission categories. ^d^Heterosexual contact with a person known to have, or to be at high risk for, HIV infection. ^e^Includes hemophilia, blood transfusion, perinatal exposure and risk factor not reported or not identified.

**Table 2:** Linkage to care among persons aged ≥13 years with HIV diagnosed during 2012-2015, by race, sex and selected characteristics – 17 US jurisdictionsa.

**Characteristic**	**Total**	**Linked to care^b^ for persons diagnosed in the year**								**Trends from 2012 - 2015**			
		**2012**		**2013**		**2014**		**2015**		**EAPC**	**95% CI**		***P*-value**
		**(N=20,876)**		**(N=19,887)**		**(N=20,467)**		**(N=19,944)**					
		**No.**	**(%)**	**No.**	**(%)**	**No.**	**(%)**	**No.**	**(%)**		**Lower**	**Upper**	
**Black Males**													
**Age group at diagnosis**													
13-24	8,002	1,442	70.5	1,482	74.7	1,541	75.0	1,511	78.8	3.4	1.1	5.8	0.004
25-34	7,513	1,242	73.5	1,356	75.4	1,591	80.4	1,594	77.9	2.3	0.0	4.7	0.054
35-44	3,612	747	75.9	705	78.4	721	82.2	683	80.2	2.2	-1.1	5.5	0.198
45-54	3,090	692	76.8	631	79.4	571	82	569	81.5	2.2	-1.3	5.8	0.227
55+	1,890	384	79.7	374	78.2	404	82.6	364	82.5	1.6	-2.9	6.3	0.485
**Stage at diagnosis**													
HIV infection stage 3(AIDS)	4,808	1,304	98.4	1,171	99.3	1,208	99.5	1,085	99.5	0.4	-2.1	3.0	0.774
Not known to be HIV infection stage 3	19,299	3,203	67.1	3,377	70.7	3,620	74.1	3,636	74.8	3.8	2.2	5.3	<.001
**Transmission Category^c^**													
Male-to-male sexual contact	19,012	3,463	73.9	3,562	76.7	3,876	79.1	3,790	79.3	2.4	1.0	3.9	0.001
Injection drug use	1,212	284	75.2	216	72.2	204	76.6	202	75.4	0.6	-5	6.5	0.841
Male-to-male sexual contact and injection drug use	603	102	69.0	116	67.3	124	79.5	95	75.6	4.8	-3.9	14.3	0.292
Heterosexual contact**^d^**	3,229	647	74.1	644	77.9	615	81.0	625	81.1	3.1	-0.4	6.8	0.08
Other**^e^**	51	11	65.6	10	89.2	9	72.5	8	72.2	1.6	-23	34.1	0.911
**Subtotal**	24,107	4,507	73.9	4,548	76.4	4,828	79.2	4,721	79.3	2.5	1.2	3.8	<.001
**Black Females**													
**Age group at diagnosis**													
13-24	1,479	311	76.4	307	80.4	279	79.9	286	83.9	2.8	-2.3	8.1	0.289
25-34	2,364	512	80.8	470	82.6	493	84	480	83.6	1.2	-2.7	5.3	0.541
35-44	2,111	448	79.3	441	83.8	455	84.1	400	83.5	1.7	-2.5	6.0	0.446
45-54	1,827	458	84.0	384	84.2	357	85.0	355	87.4	1.3	-3.1	5.8	0.6
55+	1,228	263	81.9	248	82.9	274	89.0	264	88.0	2.9	-2.5	8.6	0.302
**Stage at diagnosis**													
HIV infection stage 3(AIDS)	2,092	573	99.1	501	98.8	529	99.2	472	99.6	0.2	-3.6	4.1	0.932
Not known to be HIV infection stage 3	6,917	1,419	74.9	1,349	78.2	1,329	79.5	1,313	80.8	2.5	0.1	4.9	0.045
**Transmission Category^c^**													
Injection drug use	948	236	76.8	184	80.0	165	78.3	161	80.2	1.2	-5.0	7.8	0.715
Heterosexual contact**^d^**	8,017	1,746	81.1	1,653	83.2	1,689	85	1,615	85.5	1.8	-0.3	4	0.097
Other**^e^**	43	10	89.4	13	88.6	4	54.2	9	93.1	-2.1	-27.1	31.4	0.887
**Subtotal**	9,009	1,992	80.6	1,850	82.9	1,858	84.3	1,785	85.0	1.8	-0.2	3.9	0.083
**Hispanic/Latino Males**													
**Age group at diagnosis**													
13-24	4,586	864	75.0	848	79.8	940	79.5	992	83.4	3.2	0.3	6.2	0.033
25-34	7,532	1,507	80.1	1,497	80.5	1,568	81.9	1,555	82.8	1.2	-1.1	3.5	0.301
35-44	4,334	860	80.2	879	86.0	942	82.8	930	84.3	1.1	-1.8	4.1	0.459
45-54	2,519	550	84.6	512	85.3	529	85.6	547	84.0	-0.2	-3.8	3.6	0.925
55+	996	217	87.1	203	85.3	208	86.7	227	84.4	-0.8	-6.5	5.2	0.784
**Stage at diagnosis**													
HIV infection stage 3(AIDS)	4,667	1,201	98.4	1,171	99.0	1,129	98.8	1,110	99.0	0.2	-2.4	2.8	0.906
Not known to be HIV infection stage 3	15,300	2,797	73.9	2,768	76.9	3,058	77.5	3,141	79.1	2.1	0.5	3.8	0.01
**Transmission Category^c^**													
Male-to-male sexual contact	17,246	3,398	79.5	3,348	82.1	3,644	82.1	3,721	83.4	1.4	0.0	2.9	0.056
Injection drug use	782	175	77.7	153	81.3	145	81.0	145	76.6	-0.3	-7.0	6.8	0.924
Male-to-male sexual contact and injection drug use	743	178	83.7	144	79.2	145	81.6	145	84.5	0.4	-6.4	7.7	0.903
Heterosexual contact**^d^**	1,175	244	84.3	290	88.2	250	85.5	235	88.8	1.2	-4.3	7.1	0.671
Other**^e^**	21	3	63.0	4	84.8	4	83.0	5	91.4	11.2	-27.0	69.1	0.622
**Subtotal**	19,967	3,998	79.9	3,939	82.4	4,187	82.2	4,251	83.5	1.3	-0.1	2.7	0.059
**Hispanic/Latino Females**													
**Age group at diagnosis**													
13-24	465	88	75.2	86	81.1	111	83.5	87	79.8	2.2	-6.8	12.0	0.646
25-34	729	147	76.6	134	78.4	149	77.6	136	78.2	0.5	-6.6	8.2	0.886
35-44	674	149	87.1	147	86.0	153	86.4	133	85.8	-0.4	-7.5	7.2	0.914
45-54	550	115	85.8	129	87.8	112	82.4	120	90.2	0.9	-7.0	9.4	0.834
55+	366	80	84.2	76	88.4	95	95.0	75	88.2	2.3	-7.2	12.8	0.649
**Stage at diagnosis**													
HIV infection stage 3(AIDS)	699	175	97.8	174	98.3	177	98.9	164	100.0	0.7	-5.8	7.7	0.831
Not known to be HIV infection stage 3	2,085	404	76.2	398	79.0	443	79.2	387	78.7	1	-3.3	5.5	0.654
**Transmission Category^c^**													
Injection drug use	370	69	74.8	74	74.8	77	80.1	66	79.7	2.7	-7.7	14.2	0.623
Heterosexual contact**^d^**	2,408	508	82.7	497	85.5	542	84.6	484	84.7	0.6	-3.3	4.6	0.762
Other**^e^**	7	2	85.0	2	90.0	1	100.0	1	63.6	-4.3	-56.2	109.3	0.912
**Subtotal**	2,784	579	81.7	572	84.0	620	84.0	551	84.0	0.9	-2.8	4.6	0.646
**White Males**													
**Age group at diagnosis**													
13-24	2,444	483	81.6	494	84.2	548	83.3	538	88.6	2.4	-1.5	6.5	0.23
25-34	5,364	1,134	84.6	1,060	84.6	1,132	84.1	1,222	85.8	0.4	-2.2	3.0	0.789
35-44	3,896	916	85.1	831	86.6	859	87.7	799	90.8	2.1	-1.0	5.2	0.184
45-54	3,832	977	89.7	834	87.9	823	88.8	778	89.7	0.1	-2.9	3.1	0.972
55+	2,073	448	90.0	505	86.9	461	89.7	432	90.0	0.4	-3.7	4.6	0.867
**Stage at diagnosis**													
HIV infection stage 3(AIDS)	4,008	1,046	98.8	1,020	98.5	961	98.9	934	99.3	0.2	-2.6	3.0	0.898
Not known to be HIV infection stage 3	13,601	2,912	82.4	2,704	82.1	2,862	82.9	2,835	85.4	1.2	-0.5	2.9	0.156
**Transmission Category^c^**													
Male-to-male sexual contact	15,056	3,428	86.3	3,197	86.2	3,305	86.7	3,159	88.6	0.8	-0.7	2.4	0.281
Injection drug use	775	119	83.0	134	80.4	157	83.5	231	83.5	0.6	-6.1	7.8	0.859
Male-to-male sexual contact and injection drug use	1,109	262	85.4	244	87.9	224	84.7	230	88.3	0.6	-4.9	6.4	0.828
Heterosexual contact**^d^**	639	140	85.8	143	85.0	133	86.2	144	94.7	3.2	-4.2	11.2	0.406
Other**^e^**	30	9	93.8	6	79.0	4	58.6	5	96.1	-4.6	-34.0	37.8	0.802
**Subtotal**	17,609	3,958	86.1	3,724	86	3,823	86.4	3,769	88.5	0.8	-0.6	2.3	0.242
**White Females**													
**Age group at diagnosis**													
13-24	277	62	77.5	42	73.7	64	86.5	60	90.9	6.4	-5.1	19.2	0.289
25-34	623	123	81.5	108	84.4	134	81.7	145	80.6	-0.7	-7.9	7.2	0.866
35-44	555	110	79.1	113	81.3	102	81.6	125	82.2	1.2	-6.7	9.7	0.776
45-54	473	92	88.5	99	86.8	108	90.0	118	87.4	0	-8.3	8.9	0.995
55+	288	63	87.5	68	84.0	54	90.0	63	84.0	-0.6	-11.0	11.0	0.918
**Stage at diagnosis**													
HIV infection stage 3(AIDS)	509	156	98.7	121	98.4	112	100.0	116	100.0	0.5	-6.9	8.5	0.893
Not known to be HIV infection stage 3	1,707	294	75.8	309	78.0	350	81.2	395	80.3	2.1	-2.7	7	0.398
**Transmission Category^c^**													
Injection drug use	644	109	77.4	114	79.0	102	80.9	187	80.7	1.4	-5.9	9.2	0.715
Heterosexual contact**^d^**	1,560	338	84.2	315	84.6	358	86.4	322	86.2	0.9	-3.8	5.9	0.704
Other**^e^**	12	3	79.5	1	46.2	2	75.0	2	66.7	-3.2	-47.5	78.6	0.918
**Subtotal**	2,216	450	82.4	430	82.9	462	85.1	511	84.0	0.8	-3.1	5.0	0.687
**Total**	**81,174**	**16,670**	**79.9**	**16,197**	**81.4**	**16,959**	**82.9**	**16,695**	**83.7**	**1.6**	**0.9**	**2.3**	**<.001**

Abbreviations: HIV= Human Immunodeficiency Virus, EAPC=Estimated Annual Percent. Note: The denominator for each characteristic for each year are not presented. ^a^Jurisdictions include California, District of Columbia, Hawaii, Iowa, Illinois, Indiana, Louisiana, Maryland, Michigan, Missouri, New Hampshire, New York, North Dakota, South Carolina, Texas, Utah, and West Virginia. ^b^Defined as one or more CD4+ T-lymphocyte or viral load test within 1 month after HIV diagnosis. ^c^Data statistically adjusted to account for missing transmission categories. ^d^Heterosexual contact with a person known to have, or to be at high risk for, HIV infection. ^e^Includes hemophilia, blood transfusion, perinatal exposure and risk factor not reported or not identified.

**Table 3:** Receipt of care among persons aged ≥13 years living with HIV, 17 US jurisdictions^a^, 2012-2015.

**Characteristic**	**Receipt of Care^b^**											
	**2012**		**2013**		**2014**		**2015**		**Trend in 2012-2015**			
	**(N=440,375)**		**(N=451,885)**		**(N=464,461)**		**(N=477,928)**		**EAPC**	**95% CI**		***P*-value**
	**No.**	**(%)**	**No.**	**(%)**	**No.**	**(%)**	**No.**	**(%)**		**Lower**	**Upper**	
**Sex**												
Male	240,338	70.8	250,253	71.6	261,047	72.5	267,644	72.1	0.7	0.5	0.8	<.001
Female	72,100	71.5	73,956	72.2	75,582	72.4	77,191	72.4	0.4	0.1	0.7	.011
**Race/Ethnicity**												
Black/African American	117,215	68.2	122,090	69.4	126,963	69.9	130,784	69.8	0.8	0.5	1.1	<.001
Hispanic/Latino	75,236	68.9	78,513	69.3	82,354	70.2	85,046	69.8	0.5	0.2	0.8	<.001
White	98,207	74.7	101,076	75.3	103,959	76.4	105,162	75.7	0.5	0.3	0.8	<.001
Other	21,780	78.8	22,530	79.4	23,353	79.7	23,843	79.4	0.3	-0.3	0.8	.379
**Age group at diagnosis**												
13-24	10,941	70.2	11,546	72.5	11,819	74.5	11,753	75.2	2.4	1.5	3.2	<.001
25-34	42,941	68.9	45,144	70.4	48,086	71.8	50,639	72.5	1.7	1.3	2.1	<.001
35-44	76,771	70.2	74,827	71.0	73,406	71.6	71,621	71.4	0.6	0.3	0.9	<.001
45-54	114,184	72.7	116,821	73.3	118,737	73.8	118,052	73.2	0.2	0	0.5	.058
55+	67,601	70.3	75,871	70.8	84,581	71.5	92,770	71.0	0.3	0	0.6	.039
**Stage at diagnosis**												
HIV infection stage 3(AIDS)	77,369	70.8	79,592	71.5	81,523	72.0	82,732	71.7	0.4	0.1	0.7	.006
Not known to be HIV infection stage 3	235,069	71.0	244,617	71.8	255,106	72.6	262,103	72.3	0.6	0.5	0.8	<.001
**Transmission Category^c^**												
Male-to-male sexual contact	172,781	72.7	181,665	73.4	191,919	74.4	198,957	73.9	0.6	0.4	0.8	<.001
Injection drug use	44,880	63.9	44,843	64.6	44,350	64.6	43,421	63.9	0	-0.4	0.4	.926
Male-to-male sexual contact and injection drug use	21,469	75.3	21,684	76.5	21,828	77.2	21,607	76.6	0.6	0	1.2	.037
Heterosexual contact**^d^**	72,026	70.6	74,735	71.4	77,251	71.7	79,574	71.8	0.5	0.2	0.8	.001
Other**^e^**	1,282	68.8	1,283	69.4	1,281	69.8	1,276	69.0	0.1	-2.3	2.6	.912
**Total**	**312,438**	**70.9**	**324,209**	**71.7**	**336,629**	**72.5**	**344,835**	**72.2**	**0.6**	**0.4**	**0.8**	**<.001**

Abbreviations: HIV= Human Immunodeficiency Virus, EAPC=Estimated Annual Percent Change. Note: The denominator for each characteristic for each year are not presented. ^a^Jurisdictions include California, District of Columbia, Hawaii, Iowa, Illinois, Indiana, Louisiana, Maryland, Michigan, Missouri, New Hampshire, New York, North Dakota, South Carolina, Texas, Utah, and West Virginia. ^b^Defined as one or more CD4+ T-lymphocyte or viral load test performed during the outcome year. ^c^Data statistically adjusted to account for missing transmission categories. ^d^Heterosexual contact with a person known to have, or to be at high risk for, HIV infection. ^e^Includes hemophilia, blood transfusion, perinatal exposure and risk factor not reported or not identified.

**Table 4:** Receipt of care among persons aged ≥13 years living with HIV in 17 US jurisdictions^a^ by race, sex and selected characteristics, 2012-2015

**Characteristic**	**Receipt of Care^b^**											
	**2012**		**2013**		**2014**		**2015**		**Trend in 2012-2015**			
	**(N=440,375)**		**(N=451,885)**		**(N=464,461)**		**(N=477,928)**		**EAPC**	**95% CI**		***P*-value**
	**No.**	**(%)**	**No.**	**(%)**	**No.**	**(%)**	**No.**	**(%)**		**Lower**	**Upper**	
**Black Male**												
**Age group at diagnosis**												
13-24	4,694	68.3	5,059	70.8	5,269	73.0	5,216	73.4	2.5	1.2	3.7	<.001
25-34	12,119	66.5	13,252	68.5	14,932	70.4	16,586	71.3	2.3	1.6	3.1	<.001
35-44	16,430	66.9	16,071	68.4	15,972	69.4	15,772	69.5	1.3	0.6	2.0	<.001
45-54	26,489	68.4	27,001	70.0	27,228	70.3	26,736	69.8	0.6	0.1	1.2	.018
55+	16,234	64.6	18,275	65.2	20,111	65.3	21,898	64.8	0.1	-0.6	0.7	.832
**Stage at diagnosis**												
HIV infection stage 3(AIDS)	19,669	69.2	20,250	70.4	20,732	70.8	20,973	70.3	0.5	-0.1	1.1	.101
Not known to be HIV infection stage 3	56,297	66.2	59,408	67.6	62,780	68.5	65,235	68.4	1.1	0.8	1.5	<.001
**Transmission Category^c^**												
Male-to-male sexual contact	45,019	69.0	48,073	70.2	51,704	71.1	54,684	71.1	1.0	0.6	1.4	<.001
Injection drug use	12,834	59.5	13,004	61.3	12,738	61.0	12,319	59.9	0.2	-0.6	1.0	.650
Male-to-male sexual contact and injection drug use	6,359	71.8	6,407	73.8	6,462	74.8	6,332	73.5	0.8	-0.3	1.9	.135
Heterosexual contact**^d^**	11,570	66.1	11,987	67.0	12,413	67.5	12,680	67.4	0.7	-0.1	1.5	.108
Other**^e^**	184	63.2	187	63.2	195	64.5	193	62.2	-0.3	-6.4	6.3	.930
**Black Female**												
**Age group at diagnosis**												
13-24	1,477	69.8	1,412	72.3	1,341	71.7	1,266	74.3	1.8	-0.6	4.3	.133
25-34	6,182	66.8	6,012	67.6	5,845	67.8	5,804	68.7	0.9	-0.2	2.0	.127
35-44	11,461	68.2	11,460	69.3	11,365	69.9	11,319	70.3	1.0	0.2	1.8	.020
45-54	13,997	73.3	14,297	73.5	14,582	73.4	14,736	73.0	-0.1	-0.8	0.6	.774
55+	8,132	72.8	9,251	73.4	10,318	73.5	11,451	73.2	0.2	-0.7	1.0	.738
**Stage at diagnosis**												
HIV infection stage 3(AIDS)	9,540	73.4	9,758	74.0	9,948	74.3	10,141	74.1	0.3	-0.5	1.2	.459
Not known to be HIV infection stage 3	31,709	69.8	32,674	70.6	33,503	70.9	34,435	71.1	0.6	0.1	1.1	.012
**Transmission Category^c^**												
Injection drug use	10,264	69.1	10,221	69.9	10,102	69.9	9,931	69.3	0.1	-0.8	1.0	.832
Heterosexual contact**^d^**	30,771	71.1	31,982	71.8	33,119	72.1	34,414	72.5	0.7	0.2	1.2	.008
Other**^e^**	214	71.8	230	75.3	230	74.0	231	73.0	0.3	-5.4	6.3	.918
**Hispanic/Latino Male**												
**Age group at diagnosis**												
13-24	2,183	70.8	2,422	73.6	2,571	77.7	2,676	77.8	3.3	1.5	5.2	<.001
25-34	10,254	67.6	10,952	68.9	11,973	71.5	12,631	72.5	2.5	1.7	3.3	<.001
35-44	17,365	67.8	17,085	67.9	17,026	68.7	16,973	68.6	0.5	-0.2	1.1	.167
45-54	19,947	69.0	21,062	69.5	22,025	69.7	22,554	69.1	0.0	-0.6	0.6	.879
55+	9,694	65.0	10,874	65.0	12,219	65.5	13,500	65.0	0.1	-0.8	0.9	.892
**Stage at diagnosis**												
HIV infection stage 3(AIDS)	16,836	64.6	17,505	65.2	18,097	65.6	18,544	65.5	0.5	-0.2	1.2	.142
Not known to be HIV infection stage 3	42,607	69.2	44,890	69.5	47,717	70.7	49,790	70.4	0.7	0.3	1.1	.001
**Transmission Category^c^**												
Male-to-male sexual contact	42,691	69.9	45,560	70.5	48,849	71.7	51,470	71.5	0.8	0.4	1.2	<.001
Injection drug use	7,210	56.4	7,068	55.7	7,026	55.7	6,861	54.8	-0.9	-1.9	0.2	.106
Male-to-male sexual contact and injection drug use	4,915	73.1	5,004	74.0	4,999	73.8	4,975	73.3	0.0	-1.2	1.3	.971
Heterosexual contact**^d^**	4,495	64.8	4,627	65.1	4,806	65.5	4,900	64.9	0.1	-1.2	1.4	.876
Other**^e^**	133	63.4	136	64.4	134	64.4	127	61.9	-0.7	-8.0	7.2	.863
**Hispanic/Latino Female**												
**Age group at diagnosis**												
13-24	406	70.2	381	70.4	372	74.3	348	73.4	1.9	-2.6	6.6	.417
25-34	2,139	69.4	2,065	70.2	1,978	70.2	1,923	70.0	0.3	-1.7	2.2	.791
35-44	4,262	71.9	4,228	72.5	4,177	73.3	3,993	72.4	0.4	-1.0	1.7	.611
45-54	5,553	75.5	5,640	75.5	5,771	76.0	5,759	75.5	0.1	-1.1	1.3	.875
55+	3,433	74.5	3,804	74.2	4,242	74.6	4,689	73.6	-0.4	-1.7	1.0	.612
**Stage at diagnosis**												
HIV infection stage 3(AIDS)	3,760	72.3	3,799	72.2	3,882	72.7	3,932	72.1	0.0	-1.4	1.4	.980
Not known to be HIV infection stage 3	12,033	73.6	12,319	74.0	12,658	74.7	12,780	74.0	0.2	-0.5	1.0	.549
**Transmission Category^c^**												
Injection drug use	4,256	71.8	4,211	71.8	4,170	71.7	4,107	71.1	-0.3	-1.6	1.1	.689
Heterosexual contact**^d^**	11,440	73.9	11,810	74.2	12,274	75.1	12,511	74.4	0.3	-0.5	1.1	.445
Other**^e^**	97	70.3	97	71.6	95	70.3	94	68.3	-1.0	-9.5	8.2	.819
**White Male**												
**Age group at diagnosis**												
13-24	1,181	74.3	1,215	75.5	1,246	76.4	1,292	77.6	1.5	-1.0	4.0	.258
25-34	7,593	74.6	8,077	76.3	8,378	76.9	8,602	76.4	0.8	-0.2	1.8	.117
35-44	18,741	75.1	17,634	75.8	16,721	76.6	15,606	75.7	0.4	-0.3	1.1	.267
45-54	36,888	76.3	37,190	76.7	37,270	77.7	36,283	76.8	0.4	-0.1	0.8	.127
55+	23,565	74.2	26,448	75.0	29,709	76.7	32,516	76.1	0.9	0.4	1.5	<.001
**Stage at diagnosis**												
HIV infection stage 3(AIDS)	20,210	74.4	20,660	74.9	21,108	75.9	21,276	75.6	0.6	0.0	1.2	.053
Not known to be HIV infection stage 3	67,758	75.6	69,904	76.3	72,216	77.5	73,023	76.6	0.6	0.2	0.9	.001
**Transmission Category^c^**												
Male-to-male sexual contact	73,065	76.0	75,358	76.6	77,922	77.8	78,872	76.9	0.5	0.1	0.8	.004
Injection drug use	3,936	62.2	4,012	63.6	4,036	64.5	4,004	63.9	1.0	-0.4	2.4	.171
Male-to-male sexual contact and injection drug use	8,210	77.4	8,318	78.3	8,428	79.7	8,406	79.6	1.0	0.0	2.0	.041
Heterosexual contact**^d^**	2,346	71.6	2,483	73.6	2,557	73.6	2,633	74.2	1.1	-0.7	2.9	.234
Other**^e^**	410	73.9	392	72.7	381	73.3	384	74.0	0.1	-4.2	4.6	.968
**White Female**												
**Age group at diagnosis**												
13-24	259	69.4	243	69.2	204	68.7	182	65.9	-1.5	-7.2	4.5	.610
25-34	1,365	67.6	1,340	68.3	1,294	68.1	1,275	68.8	0.5	-1.9	2.9	.701
35-44	2,681	68.1	2,639	69.5	2,521	69.2	2,448	69.2	0.4	-1.3	2.2	.643
45-54	3,690	70.5	3,790	71.2	3,843	71.0	3,864	71.5	0.4	-1.0	1.8	.607
55+	2,244	71.5	2,500	71.7	2,773	71.7	3,094	71.7	0.1	-1.6	1.8	.936
**Stage at diagnosis**												
HIV infection stage 3(AIDS)	2,221	71.5	2,313	73.1	2,336	72.5	2,369	73.0	0.5	-1.3	2.4	.573
Not known to be HIV infection stage 3	8,018	69.2	8,199	69.7	8,299	69.7	8,494	69.9	0.3	-0.6	1.3	.512
**Transmission Category^c^**												
Injection drug use	3,306	65.9	3,332	66.6	3,342	66.9	3,337	66.9	0.5	-1.1	2.0	.560
Heterosexual contact**^d^**	6,789	71.7	7,040	72.5	7,155	72.1	7,384	72.4	0.2	-0.8	1.3	.653
Other**^e^**	145	66.5	139	65.8	138	66.8	142	68.9	1.2	-6.0	8.9	.748
**Total**	**312,438**	**70.9**	**324,209**	**71.7**	**336,629**	**72.5**	**344,835**	**72.2**	**0.6**	**0.4**	**0.8**	**<.001**

Abbreviations: HIV= Human Immunodeficiency Virus, EAPC=Estimated Annual Percent Change. Note: The denominator for each characteristic for each year are not presented. ^a^Jurisdictions include California, District of Columbia, Hawaii, Iowa, Illinois, Indiana, Louisiana, Maryland, Michigan, Missouri, New Hampshire, New York, North Dakota, South Carolina, Texas, Utah, and West Virginia. ^b^Defined as one or more CD4+ T-lymphocyte or viral load test performed during the outcome year. ^c^Data statistically adjusted to account for missing transmission categories. ^d^Heterosexual contact with a person known to have, or to be at high risk for, HIV infection. ^e^Includes hemophilia, blood transfusion, perinatal exposure and risk factor not reported or not identified.

**Table 5:** Viral suppression among persons aged ≥13 years living with HIV, 17 US jurisdictions^a^, 2012-2015

**Characteristic**	**Viral Suppression**											
	**2012**		**2013**		**2014**		**2015**		**Trend in 2012-2015**			
	**(N=440,375)**		**(N=451,885)**		**(N=464,461)**		**(N=477,928)**		**EAPC**	**95% CI**		***P*-value**
	**No.**	**(%)**	**No.**	**(%)**	**No.**	**(%)**	**No.**	**(%)**		**Lower**	**Upper**	
**Sex**												
Male	184,330	54.3	199,908	57.2	214,601	59.6	221,923	59.8	3.3	3.1	3.5	<.001
Female	50,921	50.5	54,913	53.6	58,458	56.0	60,886	57.1	4.2	3.8	4.6	<.001
**Race/Ethnicity**												
Black/Afican American	80,058	46.6	87,808	49.9	94,951	52.3	99,078	52.9	4.3	4	4.6	<.001
Hispanic/Latino	58,732	53.8	63,416	56.0	68,760	58.6	71,787	59.0	3.2	2.9	3.6	<.001
White	80,078	60.9	85,921	64.1	90,356	66.4	92,223	66.4	3	2.7	3.3	<.001
Other	16,383	59.3	17,676	62.3	18,992	64.8	19,721	65.7	3.5	2.8	4.2	<.001
**Age group at diagnosis**												
13-24	6,201	39.8	7,200	45.2	7,911	49.9	8,246	52.8	9.8	8.6	10.9	<.001
25-34	28,504	45.8	32,115	50.1	35,666	53.3	38,058	54.5	5.9	5.4	6.4	<.001
35-44	56,423	51.6	57,282	54.3	58,184	56.8	57,243	57.1	3.5	3.1	3.9	<.001
45-54	88,692	56.5	94,059	59.0	98,182	61.0	98,479	61.0	2.7	2.4	3	<.001
55+	55,431	57.7	64,165	59.9	73,116	61.8	80,783	61.8	2.3	2	2.7	<.001
**Stage at diagnosis**												
HIV infection stage 3(AIDS)	61,281	56.1	65,045	58.4	68,374	60.4	69,991	60.6	2.7	2.3	3	<.001
Not known to be HIV infection stage 3	173,970	52.5	189,776	55.7	204,685	58.3	212,818	58.7	3.8	3.6	4	<.001
**Transmission Category^c^**												
Male-to-male sexual contact	135,257	56.9	148,554	60.0	160,922	62.4	168,400	62.6	3.2	3	3.5	<.001
Injection drug use	31,578	44.9	32,747	47.2	33,706	49.1	33,182	48.8	2.9	2.4	3.4	<.001
Male-to-male sexual contact and injection drug use	15,331	53.7	16,017	56.5	16,769	59.3	16,652	59.0	3.3	2.6	4.1	<.001
Heterosexual contact**^d^**	52,111	51.1	56,492	54.0	60,613	56.3	63,499	57.3	3.9	3.5	4.2	<.001
Other**^e^**	974	52.3	1,011	54.7	1,050	57.2	1,076	58.2	3.7	0.9	6.6	.009
**Total**	**235,251**	**53.4**	**254,821**	**56.4**	**273,059**	**58.8**	**282,809**	**59.2**	**3.5**	**3.3**	**3.7**	**<.001**

Abbreviations: HIV= Human Immunodeficiency Virus, EAPC=Estimated Annual Percent Change. Note: The denominator for each characteristic for each year are not presented. ^a^Jurisdictions include California, District of Columbia, Hawaii, Iowa, Illinois, Indiana, Louisiana, Maryland, Michigan, Missouri, New Hampshire, New York, North Dakota, South Carolina, Texas, Utah, and West Virginia. ^b^Defined as a viral load result of <200 copies/mL or, if the quantitative value was missing, a test interpretation value of “undetected”, at the time of the most recent viral load test during the outcome year. ^c^Data statistically adjusted to account for missing transmission categories. ^d^Heterosexual contact with a person known to have, or to be at high risk for, HIV infection. ^e^Includes hemophilia, blood transfusion, perinatal exposure and risk factor not reported or not identified.

**Table 6:** Viral suppression among persons aged ≥13 years living with HIV in 17 US jurisdictions by race, sex and selected characteristics, 2012-2015

**Characteristic**	**Viral Suppression**											
	**2012**		**2013**		**2014**		**2015**		**Trend in 2012-2015**			
	**(N=440,375)**		**(N=451,885)**		**(N=464,461)**		**(N=477,928)**		**EAPC**	**95% CI**		***P*-value**
	**No.**	**(%)**	**No.**	**(%)**	**No.**	**(%)**	**No.**	**(%)**		**Lower**	**Upper**	
**Black Male**												
**Age group at diagnosis**												
13-24	2,513	36.6	2,967	41.5	3,348	46.4	3,436	48.3	9.8	8.0	11.5	<.001
25-34	7,340	40.3	8,697	44.9	10,253	48.3	11,560	49.7	7.0	6.0	8.0	<.001
35-44	11,105	45.2	11,413	48.6	11,852	51.5	11,666	51.4	4.5	3.7	5.4	<.001
45-54	18,904	48.8	20,028	51.9	20,868	53.9	20,553	53.7	3.2	2.6	3.9	<.001
55+	12,388	49.3	14,368	51.3	16,204	52.6	17,856	52.8	2.3	1.5	3.0	<.001
**Stage at diagnosis**												
HIV infection stage 3(AIDS)	14,476	51.0	15,418	53.6	16,244	55.4	16,603	55.7	3.0	2.3	3.7	<.001
Not known to be HIV infection stage 3	37,774	44.4	42,055	47.9	46,281	50.5	48,468	50.8	4.6	4.1	5.0	<.001
**Transmission Category^c^**												
Male-to-male sexual contact	30,899	47.3	34,892	50.9	38,904	53.5	41,682	54.2	4.5	4.1	5.0	<.001
Injection drug use	8,850	41.0	9,267	43.7	9,451	45.2	9,040	44.0	2.5	1.5	3.4	<.001
Male-to-male sexual contact and injection drug use	4,202	47.4	4,394	50.6	4,651	53.8	4,513	52.4	3.6	2.3	5.0	<.001
Heterosexual contact**^d^**	8,182	46.8	8,800	49.2	9,379	51.0	9,699	51.6	3.3	2.4	4.3	<.001
Other**^e^**	117	40.2	120	40.6	140	46.3	137	44.3	4.3	-3.5	12.7	.288
**Black Female**												
**Age group at diagnosis**												
13-24	683	32.3	741	37.9	758	40.6	778	45.7	11.7	8.1	15.4	<.001
25-34	3,544	38.3	3,727	41.9	3,780	43.8	3,901	46.2	6.2	4.7	7.8	<.001
35-44	7,537	44.9	7,909	47.8	8,228	50.6	8,352	51.8	5.0	4.0	6.0	<.001
45-54	9,887	51.7	10,560	54.3	11,190	56.3	11,487	56.9	3.2	2.4	4.1	<.001
55+	6,157	55.1	7,398	58.7	8,470	60.3	9,489	60.6	3.0	2.0	4.1	<.001
**Stage at diagnosis**												
HIV infection stage 3(AIDS)	6,984	53.7	7,471	56.7	7,823	58.4	8,140	59.5	3.4	2.4	4.4	<.001
Not known to be HIV infection stage 3	20,824	45.8	22,864	49.4	24,603	52.0	25,867	53.4	5.2	4.6	5.8	<.001
**Transmission Category^c^**												
Injection drug use	6,693	45.1	7,062	48.3	7,318	50.6	7,323	51.1	4.3	3.2	5.4	<.001
Heterosexual contact**^d^**	20,969	48.4	23,108	51.9	24,930	54.3	26,503	55.9	4.8	4.2	5.4	<.001
Other**^e^**	146	49.1	166	54.2	178	57.3	182	57.4	5.2	-1.7	12.6	.140
**Hispanic/Latino Male**												
**Age group at diagnosis**												
13-24	1,389	45.1	1,677	51.0	1,904	57.5	2,044	59.4	9.7	7.4	12.0	.001
25-34	7,437	49.0	8,360	52.6	9,452	56.4	10,111	58.0	5.8	4.9	6.8	<.001
35-44	13,552	52.9	13,819	54.9	14,225	57.4	14,231	57.5	3.0	2.2	3.7	<.001
45-54	16,193	56.1	17,525	57.8	18,893	59.8	19,501	59.8	2.2	1.6	2.9	<.001
55+	8,258	55.4	9,455	56.5	10,909	58.5	12,037	57.9	1.6	0.8	2.5	<.001
**Stage at diagnosis**												
HIV infection stage 3(AIDS)	13,921	53.4	14,727	54.9	15,701	57.0	16,111	56.9	2.3	1.6	3.0	<.001
Not known to be HIV infection stage 3	32,908	53.4	36,109	55.9	39,682	58.8	41,813	59.1	3.5	3.1	4.0	<.001
**Transmission Category^c^**												
Male-to-male sexual contact	34,225	56.1	37,902	58.6	41,716	61.2	44,306	61.6	3.2	2.8	3.7	<.001
Injection drug use	5,439	42.6	5,462	43.1	5,662	44.9	5,526	44.1	1.5	0.3	2.7	.012
Male-to-male sexual contact and injection drug use	3,520	52.4	3,667	54.2	3,854	56.9	3,835	56.5	2.8	1.3	4.2	<.001
Heterosexual contact**^d^**	3,533	51.0	3,691	51.9	4,033	54.9	4,137	54.8	2.8	1.3	4.2	<.001
Other**^e^**	112	53.6	115	54.4	119	57.3	120	58.3	3.1	-4.9	11.9	.460
**Hispanic/Latino Female**												
**Age group at diagnosis**												
13-24	246	42.6	239	44.2	243	48.5	234	49.4	5.5	-0.3	11.7	.061
25-34	1,409	45.7	1,449	49.3	1,452	51.5	1,450	52.8	4.9	2.5	7.3	<.001
35-44	3,117	52.6	3,153	54.0	3,248	57.0	3,197	58.0	3.5	1.9	5.2	<.001
45-54	4,282	58.2	4,454	59.7	4,717	62.1	4,828	63.3	3.0	1.7	4.3	<.001
55+	2,849	61.9	3,285	64.1	3,717	65.4	4,154	65.2	1.7	0.2	3.2	.028
**Stage at diagnosis**												
HIV infection stage 3(AIDS)	2,990	57.5	3,121	59.3	3,298	61.8	3,410	62.5	2.9	1.4	4.6	<.001
Not known to be HIV infection stage 3	8,913	54.5	9,459	56.8	10,079	59.4	10,453	60.5	3.6	2.7	4.5	<.001
**Transmission Category^c^**												
Injection drug use	3,037	51.2	3,099	52.9	3,194	54.9	3,260	56.5	3.4	1.8	5.0	<.001
Heterosexual contact**^d^**	8,792	56.8	9,406	59.1	10,105	61.8	10,525	62.6	3.4	2.5	4.3	<.001
Other**^e^**	74	53.8	75	55.2	78	57.6	78	56.9	2.1	-7.6	12.9	.679
**White Male**												
**Age group at diagnosis**												
13-24	803	50.5	931	57.8	966	59.3	1,060	63.7	7.3	4.3	10.4	<.001
25-34	5,580	54.9	6,420	60.6	6,911	63.4	7,152	63.6	4.8	3.7	6.0	<.001
35-44	14,844	59.5	14,568	62.6	14,139	64.8	13,338	64.7	2.9	2.2	3.7	<.001
45-54	30,648	63.4	32,129	66.2	32,669	68.1	32,065	67.9	2.4	1.9	2.9	<.001
55+	20,434	64.3	23,543	66.8	26,875	69.4	29,606	69.3	2.5	2.0	3.1	<.001
**Stage at diagnosis**												
HIV infection stage 3(AIDS)	17,032	62.7	18,019	65.3	18,688	67.2	18,950	67.3	2.4	1.7	3.1	<.001
Not known to be HIV infection stage 3	55,277	61.6	59,572	65.0	62,872	67.4	64,271	67.4	3.0	2.7	3.4	<.001
**Transmission Category^c^**												
Male-to-male sexual contact	60,780	63.3	65,462	66.5	68,995	68.9	70,553	68.8	2.8	2.5	3.2	<.001
Injection drug use	3,024	47.8	3,171	50.3	3,252	52.0	3,210	51.3	2.5	0.9	4.1	.002
Male-to-male sexual contact and injection drug use	6,244	58.9	6,541	61.6	6,810	64.4	6,877	65.1	3.5	2.4	4.6	<.001
Heterosexual contact**^d^**	1,932	58.9	2,083	61.7	2,172	62.5	2,238	63.1	2.2	0.2	4.1	.028
Other**^e^**	329	59.4	334	61.9	332	63.7	343	66.1	3.6	-1.3	8.6	.151
**White Female**												
**Age group at diagnosis**												
13-24	147	39.4	148	42.2	132	44.4	127	46.0	5.3	-2.2	13.5	.172
25-34	907	44.9	921	47.0	965	50.8	926	49.9	4.0	1.1	7.1	.007
35-44	1,925	48.9	1,978	52.1	1,952	53.6	1,907	53.9	3.2	1.2	5.3	.002
45-54	2,926	55.9	3,116	58.5	3,265	60.3	3,274	60.6	2.7	1.1	4.3	<.001
55+	1,864	59.4	2,167	62.1	2,482	64.1	2,768	64.1	2.5	0.7	4.4	.007
**Stage at diagnosis**												
HIV infection stage 3(AIDS)	1,786	57.5	1,914	60.5	2,017	62.6	2,047	63.1	3.1	1.1	5.2	.003
Not known to be HIV infection stage 3	5,983	51.6	6,416	54.6	6,779	56.9	6,955	57.3	3.6	2.4	4.7	<.001
**Transmission Category^c^**												
Injection drug use	2,359	47.1	2,518	50.4	2,625	52.5	2,620	52.5	3.7	1.9	5.6	<.001
Heterosexual contact**^d^**	5,294	55.9	5,689	58.6	6,055	61.0	6,255	61.4	3.2	2.0	4.4	<.001
Other**^e^**	116	53.4	123	58.0	116	56.2	127	61.6	4.1	-3.9	12.7	.327
**Total**	**235,251**	**53.4**	**254,821**	**56.4**	**273,059**	**58.8**	**282,809**	**59.2**	**3.5**	**3.3**	**3.7**	**<.001**

Abbreviations: HIV= Human Immunodeficiency Virus, EAPC=Estimated Annual Percent Change. Note: The denominator for each characteristic for each year are not presented. ^a^Jurisdictions include California, District of Columbia, Hawaii, Iowa, Illinois, Indiana, Louisiana, Maryland, Michigan, Missouri, New Hampshire, New York, North Dakota, South Carolina, Texas, Utah, and West Virginia. ^b^Defined as a viral load result of <200 copies/mL or, if the quantitative value was missing, a test interpretation value of “undetected”, at the time of the most recent viral load test during the outcome year. ^c^Data statistically adjusted to account for missing transmission categories. ^d^Heterosexual contact with a person known to have, or to be at high risk for, HIV infection. ^e^Includes hemophilia, blood transfusion, perinatal exposure and risk factor not reported or not identified.

## References

[1] Centers for Disease Control and Prevention https://www.cdc.gov/hiv/pdf/library/reports/surveillance/cdc-hiv-surveillance-report-2015-vol-27.pdf.

[2] Mugavero M.J., Amico K.R., Horn T., Thompson M.A. (2013). The state of engagement in HIV care in the United States: From cascade to continuum to control.. Clin. Infect. Dis..

[3] Office of National AIDS Policy https://files.hiv.gov/s3fs-public/nhas-update.pdf.

[4] Office of National AIDS Policy https://files.hiv.gov/s3fs-public/nhas-indicators-supplement-dec-2016.pdf.

[5] Hall H.I., Gray K.M., Tang T., Li J., Shouse L., Mermin J. (2012). Retention in care of adults and adolescents living with HIV in 13 U.S. areas.. J. Acquir. Immune Defic. Syndr..

[6] McCree D.H., Sutton M., Bradley E., Harris N. (2017). Changes in the disparity of HIV diagnosis rates among black women-United States, 2010-2014.. MMWR Morb. Mortal. Wkly. Rep..

[7] Colasanti J., Stahl N., Farber E.W., Del Rio C., Armstrong W.S. (2017). An exploratory study to assess individual and structural level barriers associated with poor retention and re-engagement in care among persons living with HIV/AIDS.. J. Acquir. Immune Defic. Syndr..

[8] Penman-Aguilar A., Harrison K.M., Dean H.D. (2013). Identifying the root causes of health inequities: Reflections on the 2011 National Center for HIV/AIDS, Viral Hepatitis, STD, and TB Prevention health equity symposium.. Public Health Rep..

[9] Centers for Disease Control and Prevention http://www.cdc.gov/hiv/pdf/prevention_ongoing_surveillance_systems.pdf.

[10] Karch D.L., Chen M., Tang T. (2014). Evaluation of the national human immunodeficiency virus surveillance system for the 2011 diagnosis year.. J. Public Health Manag. Pract..

[11] Centers for Disease Control and Prevention http://www.cdc.gov/hiv/pdf/library/reports/surveillance/cdc-hiv-surveillance-supplemental-report-vol-21-4.pdf.

[12] Centers for Disease Control and Prevention Social determinants of health and selected HIV care outcomes among adults with diagnosed HIV in 32 states and the District of Columbia, 2014..

[13] Johnson A.S., Hall H.I., Hu X., Lansky A., Holtgrave D.R., Mermin J. (2014). Trends in diagnoses of HIV infection in the United States, 2002-2011.. JAMA.

[14] Centers for Disease Control and Prevention (2014). Revised surveillance case definition for HIV infection-United States.

[15] Harrison K.M., Kajese T., Hall H.I., Song R. (2008). Risk factor redistribution of the national HIV/AIDS surveillance data: An alternative approach.. Public Health Rep..

[16] Bradley H., Mattson C., Beer L., Huang P., Shouse R.L. http://www.croiconference.org/sessions/increased-hiv-viral-suppression-among-us-adults-receiving-medical-care-2009-2013.

[17] Centers for Disease Control and Prevention

[18] Aibibula W., Cox J., Hamelin A.M., McLinden T., Klein M.B., Brassard P. (2017). Association between food insecurity and HIV viral suppression: A systematic review and meta-analysis.. AIDS Behav..

[19] Tobias C.R., Cunningham W., Cabral H.D., Cunningham C.O., Eldred L., Naar-King S., Bradford J., Sohler N.L., Wong M.D., Drainoni M.L. (2007). Living with HIV but without medical care: Barriers to engagement.. AIDS Patient Care STDS.

[20] Horstmann E., Brown J., Islam F., Buck J., Agins B.D. (2010). Retaining HIV-infected patients in care: Where are we? Where do we go from here?. Clin. Infect. Dis..

[21] Wohl A.R., Galvan F.H., Myers H.F., Garland W., George S., Witt M., Cadden J., Operskalski E., Jordan W., Carpio F., Lee M.L. (2011). Do social support, stress, disclosure and stigma influence retention in HIV care for Latino and African American men who have sex with men and women?. AIDS Behav..

[22] Torian L.V., Wiewel E.W., Liu K.L., Sackoff J.E., Frieden T.R. (2008). Risk factors for delayed initiation of medical care after diagnosis of human immunodeficiency virus.. Arch. Intern. Med..

[23] Bulsara SM, Wainberg ML, Newton-John TRO Predictors of adult retention in HIV care: A systematic review..

[24] Centers for Disease Control and Prevention Compendium of evidence-based intervention and best practices for HIV prevention. Available from http://www.cdc.gov/hiv/research/interventionresearch/ compendium/lrc/index.html.

[25] Greenberg A.E., Purcell D.W., Gordon C.M., Barasky R.J., del Rio C. (2015). Addressing the challenges of the HIV continuum of care in high-prevalence cities in the United States.. J. Acquir. Immune Defic. Syndr..

[26] Grant R.M., Lama J.R., Anderson P.L., McMahan V., Liu A.Y., Vargas L., Goicochea P., Casapía M., Guanira-Carranza J.V., Ramirez-Cardich M.E., Montoya-Herrera O., Fernández T., Veloso V.G., Buchbinder S.P., Chariyalertsak S., Schechter M., Bekker L.G., Mayer K.H., Kallás E.G., Amico K.R., Mulligan K., Bushman L.R., Hance R.J., Ganoza C., Defechereux P., Postle B., Wang F., McConnell J.J., Zheng J.H., Lee J., Rooney J.F., Jaffe H.S., Martinez A.I., Burns D.N., Glidden D.V., iPrEx Study Team (2010). Preexposure chemoprophylaxis for HIV prevention in men who have sex with men.. N. Engl. J. Med..

[27] Dailey A.F., Johnson A.S., Wu B. (2017). HIV care outcomes among blacks with diagnosed HIV-United States, 2014.. MMWR Morb. Mortal. Wkly. Rep..

[28] Office of National AIDS Policy https://files.hiv.gov/s3fs-public/nhas.pdf.

[29] Crepaz N., Tang T., Marks G., Hall H.I. http://www.croiconference.org/sessions/viral-load-dynamics-among-persons-diagnosed-hiv-united-states-2014.

